# Data on social media use related to age, gender and trust constructs of integrity, competence, concern, benevolence and identification

**DOI:** 10.1016/j.dib.2018.03.065

**Published:** 2018-03-21

**Authors:** Gillian Warner-Søderholm, Andy Bertsch, Annika Søderholm

**Affiliations:** a BI Norwegian Business School, Oslo, Norway; b Minot State University, Minot, ND, USA

## Abstract

This article contains data collected from self-report surveys of respondents to measure 1) social media usage, 2) age, 3) gender and 4) trust, measured within five major trust constructs of a) Integrity, b) Competence, c) Concern, d) Benevolence and e) Identification. The data includes all instruments used, SPSS syntax, the raw survey data and descriptive statistics from the analyses. Raw data was entered into SPSS software and scrubbed using appropriate techniques in order to prepare the data for analysis. We believe that our dataset and instrument may give important insights related to computers in human behavior, and predicting trust antecedents in social media use such as age, gender, number of hour online and choice of content provider. We have also created a parsimonious five factor trust instrument developed from the extant literature for future research. Hence, this newly developed trust instrument can be used to measure trust not only in social media, but also in other areas such as healthcare, economics and investor relations, CSR, management and education. Moreover, the survey items developed to measure social media use are concise and may be applied to measure social media use in other contexts such as national cultural differences, marketing and tourism. For interpretation and discussion of the data and constructs, please see original article entitled “Who trusts social media” (Warner-Søderholm et al., 2018) [1].

**Specifications table**

**Table:** 

Subject area	Communication and management
More specific subject areas	Social media analysis studies
Type of data	Text files and SPSS file, instrument, survey data
How data was acquired	Survey, analytics, self-report questionnaires
Data format	Raw data and SPSS data
Experimental factors	
Experimental features	
Data source location	Minot, ND, USA and Oslo, Norway.
Data accessibility	Data are available with this article

**Value of the data**

• The data may be utilized to build social media analytics for larger studies within new research fields such as leadership, followership, culture, negotiations, board of directors, entrepreneurship, healthcare, marketing etc.
• The data expands on the results reported in the original study, with additional data related to what respondents documented they specifically share on social media and their political views.
• Provides raw data available for comparison with other survey data.
• The instrument may help marketers estimate the social media use patterns of each user, classify patterns of usage, and of Benevolence, Integrity, Competence, Identification, and Concern levels of trust in consumer segments to ensure customer expectations are met.
• The dataset can be used in new research – it can be expanded to include new respondent group data to explore regional differences / demographic differences / national differences / international differences etc., in a new comparative study of social media use. In addition, data can be extracted to apply in new studies of trust in a multi-regional empirical setting.

## 1 Data

The data presented in this article document the responses to the newly revised 25 trust construct measurement items from 214 respondents from a convenience sample of university students and faculty (see Supplementary file in the Appendix). Data for 3 items developed to measure contemporary social media use are also presented along with 3 demographic items used in the study. In addition, the article provides copies of the instruments that were used to gather the data and the SPSS syntax, making the instrument freely available for future academic and business research. The subsequent raw data, as well as descriptive statistics can be added to new datasets. The responses may also be used to seek correlation or may be selected based on subsets to test new hypotheses in future studies.

## 2 Experimental design, materials and methods

**Fig. 1:**
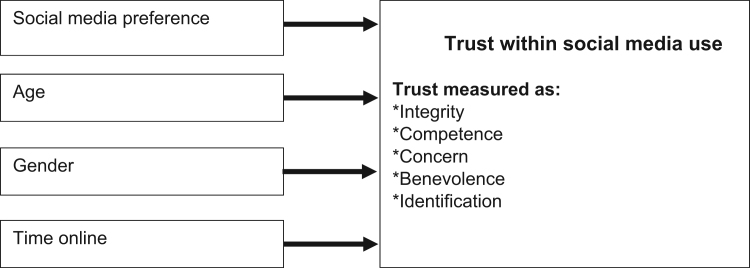
Conceptual model investigating social media and newsfeeds use and trust.

The study utilized a questionnaire-based survey instrument to collect data via a paper-based and online instrument. Coding the data in numerical form for appropriate statistical analyses was carried out via Likert scale 1–5 coding applied to the trust items (1 = strongly agree, 5 = strongly disagree). The instrument employed in this study was an amalgamation from McKnight et al. [2], Mayer and Davis [3], and Shockley-Zalabak et al. [4]. Specifically, items were utilized from McKnight, Choudhury, and Kacmar [2] to measure Benevolence (5 items) and Competence (5 items). From Mayer and Davis [3] we utilized items to measure Integrity (5 items). Survey questions related to the constructs Identification (5 items) and Concern (5 items), were utilized from Shockley-Zalabak et al. [4]. The questions used to measure relevant social media use were adapted from www.marketest.co.uk. Additionally, to summarize our approach in support of our methods, this study was exploratory in nature, hence we sought to explore relationships across various variables that have been discussed in the literature review, yet which had not been explored empirically, nor clearly defined in the literature prior to this study. Further, we sought to discover new information and new relationships; therefore, we selected a sample based on convenience, an important allowance within exploratory research. Our sample was drawn from an international sample pool of university business students and faculty. We employed an exploratory research design therefore, consisting of borrowed trust measure items, convenience sampling, and a positivistic, quantitative methodology and this is fully described in the article [1]. The study can consequently be visualized in the following conceptual model, depicted in Fig. 1 below:

## Declarations of interest

None.
